# The measurement of volume change by capillary dilatometry

**DOI:** 10.1002/pro.3626

**Published:** 2019-04-29

**Authors:** Peter C. Kahn

**Affiliations:** ^1^ Department of Biochemistry and Microbiology Rutgers University New Brunswick New Jersey 08901

**Keywords:** dilatometry, volume change, hydration, hydrophobicity, electrostriction

## Abstract

Capillary dilatometry enables direct measurement of changes in volume, an extensive thermodynamic property. The results provide insight into the changes in hydration that occur upon protein folding, ligand binding, and the interactions of proteins with nucleic acids and other cellular components. Often the entropy change arising from release of hydrating solvent provides the main driving force of a binding reaction. For technical reasons, though, capillary dilatometry has not been as widely used in protein biochemistry and biophysics as other methods such as calorimetry. Described here are simple apparatus and simple methods, which bring the technique within the capacity of any laboratory. Even very simple results are shown to have implications for macromolecular‐based phenomena. Protein examples are described.

**Abbreviations**
ICT: international critical tables
HPLC: high‐pressure liquid chromatography
GuHCl: guanidine hydrochloride
CD: circular dichroism

## Introduction

Biochemical processes are often attended by changes in volume. Most often, these arise from changes in hydration as water molecules are expelled from the biomolecular surface into the bulk solution or as exposure of molecular surface to the solvent leads to increased hydration. Protein folding and unfolding, subunit association and ligand binding are good examples.^1^, ^2^, ^3^, ^4^, ^5^, ^6^, ^7^, ^8^, ^9^, ^10^ Hydrogen ions, in particular, have dramatic effects, as the dissociation of a proton from a carboxyl group, for example, occurs with a Δ*V* of −11 mL/mol of carboxylate formed.^4^ Interactions involving nucleic acids also lead to volume changes for the same reasons as for proteins. The helix to coil transitions of duplex nucleic acids, for example, have been well characterzed,^11^ as have the hydration changes arising from the intercalation of DNA binding drugs into the double helix.^12^ Chalikian and Breslauer^13^ and Chalikian and Macgregor^14^ have provided good reviews of the nucleic acid work. It is interesting that the volumetric work on nucleic acids was done by high precision measurements of density using a vibrating tube densimeter, a technique, which is discussed below.

Hydration driven volume changes occur primarily because the density of water that hydrates biomolecular surfaces differs from that of bulk water. Protein unfolding exposes to the solvent previously buried surface, a large fraction of which is aliphatic. The exposure of aliphatic surface leads to a volume decrease, which is consistent with the idea that the density of the water hydrating the exposed aliphatic groups exceeds that of bulk water (e.g., Refs. 15, 16, 17). The formation or loss of packing defects also leads to volume changes.^1^, ^2^, ^18^, ^19^ However, Mitra et al.,^18^ it should be noted, interpret their pressure studies to indicate that hydrating water has a lower density than bulk water.

Although the measurement of volume changes in biochemical reactions has received attention as indicated above, the potential contribution to understanding the roles of hydrating water has yet to be fully realized, largely due to technical challenges. Kupke^19^ and Chalikian^10^ have reviewed methods for volumetric measurements. Offered here is a simplification of classical apparatus and simple experimental procedures as well as some illustrative results.

## Results

**Figure 1:**
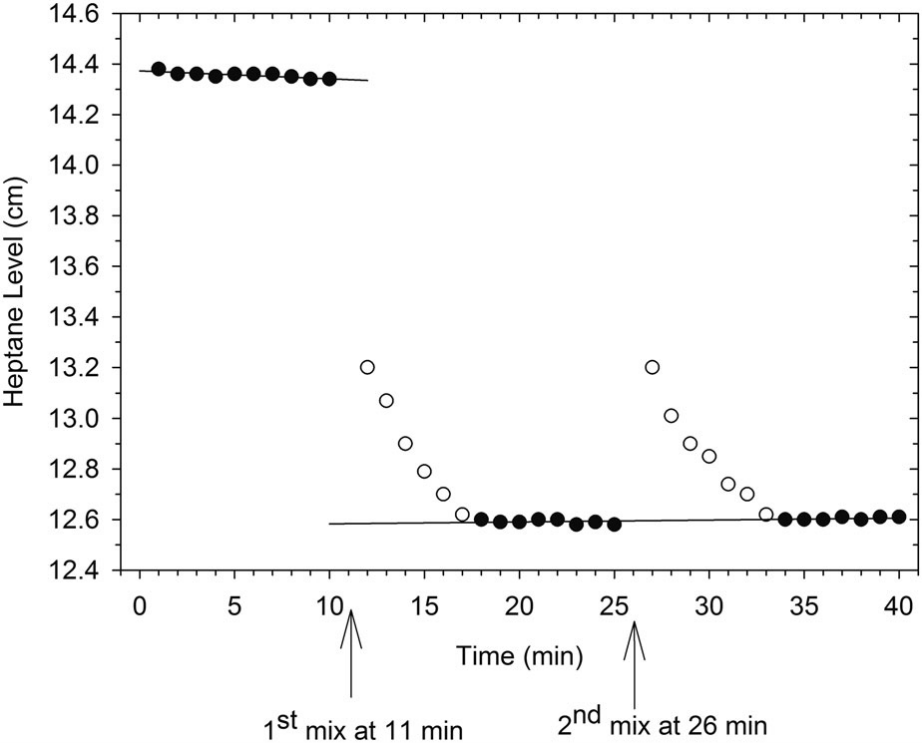
Standard KCl dilution experiment. Mixing times at 11 and 26 min are indicated by arrows. Least squares lines premixing and postmixing are shown. Data used for least squares lines are filled circles.

A KCl dilution experiment as described in the Methods is shown in Figure 1. The experiment is used as a training exercise to teach the technique. After thermal equilibration and adjustment of the meniscus (see Methods), readings at 1 min intervals began at time zero. Mixing was done after the 10 min reading and resumed at minute 12. The time of mixing was therefore taken to be minute 11. From the least squares line fitted to the premixing heptane level, the height at 11 min was found. There being no appreciable volume change after the second mixing at 26 min, a single least squares line was fitted to the data given by the postmixing filled circles in Figure 1. Extrapolation of the premixing and postmixing lines to 11 min yields the change in meniscus level, −1.754 cm. The capillary used measures 0.3298 μL/cm change in heptane level, making the volume change −0.58 μL. From the data in the International Critical Tables^20^ (ICT) this dilution should yield a volume change of −0.61 μL. The Δ*V* of −0.58 μL is 95.1% of this value. Most of the small difference is probably due to the fact that the KCl, although not particularly hygroscopic, was not dried to constant weight. Once people have done three *consecutive* experiments whose outcomes are within 15% of the theoretical value, they are ready to work with proteins.

Note in Figure 1 the slight negative slope in the premixing meniscus level. Slopes—positive or negative—occur occasionally and are probably the result of small leaks. As long as they are linear, the short extrapolation (one or a few minutes) to the time of mixing will not introduce significant error. In our location, the summer time laboratory temperature is high enough that the constant temperature bath sometimes cannot handle the heat load. When linear extrapolation is inadequate, good results can usually be restored by filling the bath surface with ping‐pong balls. They are mobile enough not to interfere with mixing, and they add a layer of insulation.

## Discussion

After each mixing in Figure 1 the meniscus falls slowly over several minutes before stabilizing at the postmixing level. With the constant temperature bath at 20°C, simply placing the hand in the bath near the dilatometer will provide enough heat to drive the meniscus up. Handling the dilatometer in order to mix the contents adds even more heat. This additional heat takes some time to dissipate. The useful data in this experiment are the difference in meniscus heights before mixing and when the postmixing level has stabilized.

### *Why is the volume change in this case negative?*

Ions in aqueous solution are surrounded by water molecules, which are pulled in toward the ion by its electrostatic field. These waters are closer to one another than would be the case in the pure solvent. This electrostatically driven contraction in volume is called electrostriction.^21^, ^22^ The negative volume change of KCl dilution arises from increased electrostriction. Before dilution, the hydration spheres of the ions overlap. Some water molecules are shared between more than one ion. When the solution is diluted, the ions move further apart, their hydration shells overlap less, and each ion acquires additional waters to its own shell. The additional electrostriction of these waters yields the volume change. There are implications of this observation for solvation changes to the ionic groups of proteins due to conformational changes and ligand binding. Ionic groups shift in their relative positions, and when they do so their hydration will change. It should be noted also that electrostricted water has a lower entropy than bulk water. This is for two reasons. The interaction between the ion and the surrounding water restricts the motional freedom of the water as does restriction to a smaller volume than in bulk solvent. This will contribute to the thermodynamics of the process. Consider, for example, a conformational change in which the distance between two solvent exposed ionically charged side chains that are initially close to one another becomes larger. As with the ions of KCl, each would acquire more water of hydration whose volume and entropy would both decrease. These contributions may be balanced by others or they may be reinforced. Any full analysis of the thermodynamics must take them into account.

**Figure 2:**
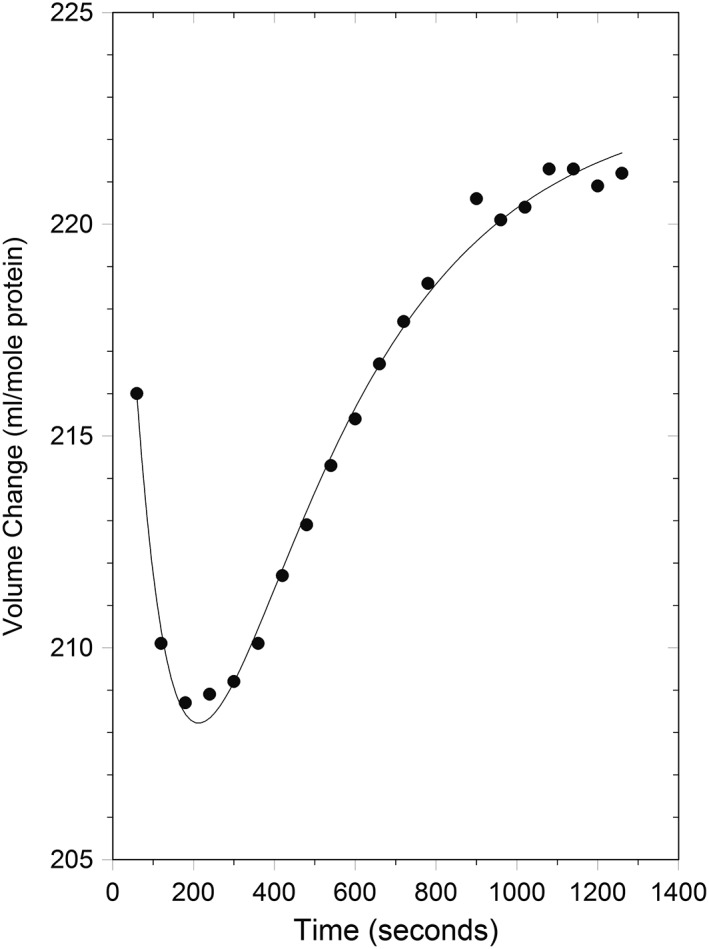
Refolding kinetics of ribonuclease A (Fig. 1 from Ref. 1). The data are the average of three experiments. Denaturing conditions: 3*M* GuHCI, 40 m*M* histidine, pH 2. Renaturing conditions: 2.5*M* GuHCI, 33 m*M* histidine, pH 6.5–7.

When a reaction involves slow kinetics, it can be followed by dilatometry. The slow refolding of ribonuclease A shown in Figure 2 1 is an example. The protein was unfolded by dialysis against a pH 2 buffer containing 3*M* guanidine hydrochloride (GuHCl). It was mixed in the dilatometer with sufficient NaOH to raise the pH to 6.5 and dilute the GuHCl to 2.5*M*, which brings about refolding. The authors were able to deconvolute the data into three phases: a fast volume rise, which was over in the dead time of the mixing (1–2 min), a slow decrease in volume, which was finished in approximately 200 sec, and an even slower rise in volume that lasted to more than 1200 sec. For each the equilibrium volume change and for the slow stages, the time constants were obtained. To correlate the volumetric measurements with structural changes, parallel kinetic circular dichroism (CD) experiments were performed using the same dilution protocol. The ellipticity at 222 and 276 nm was followed to observe secondary and tertiary structural changes, respectively. In agreement with published spectroscopic work, the CD showed a single first order process even though the volumetric data demonstrated two concurrent, distinguishable slow events. The rapid initial rise in volume was attributed to structural collapse of the unfolded form to a more compact but non‐native form. The slow volume decrease was shown to be due primarily to global conformational effects of *cis‐trans* proline isomerization, while the slower volume rise arose from the “XY” process first described by Lin and Brandts.^23^, ^24^, ^25^, ^26^ This transition is between unknown initial and final conformations, hence X and Y. It was previously detectable only by absorbance and fluorescence kinetics. The CD results also indicate that both slow processes involve backbone rearrangements, which are not, therefore, complete in the fast initial phase.

The jump in pH removes protons from the protein's titratable groups. After subtracting the control for titration of the buffer, the authors were able to estimate the volumetric contribution of deprotonating the protein itself. A positive volume change not attributable to titration *per se* remained, attributable primarily to hydrophobic collapse, which entailed expulsion of water of hydration from the protein surface as it folded. The authors estimate that ~240 water molecules were involved but they pointed out that that number is probably on the low side. Finally, the slow volume rise was attributed primarily to the formation of packing defects within the protein in the late stages of folding as the conformation made its final adjustments. These experiments were the first use of volumetric experiments as a probe of protein folding kinetics.

**Figure 3:**
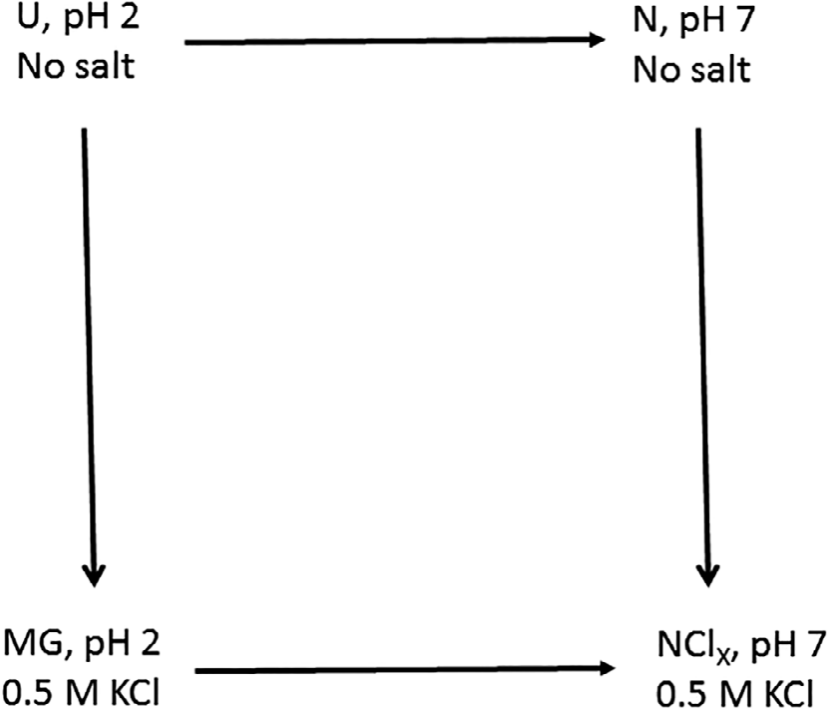
The logic of the molten globule transition measurements for cytochrome c. Simplified from Figure 1 of Ref. 2.

The molten globule (MG) transitions of cytochrome c also illustrate the application of dilatometry to protein work.^2^ The protein unfolds when the pH is reduced from 7 to 2 in the absence of added salt. Addition to the unfolded (U) form of 0.5*M* KCl at pH 2 yields a collapse to a non‐native form whose secondary structure is approximately the same as that of native (N) but whose tertiary structure fluctuates; the tertiary structure is said to be melted, and the conformational state is therefore called the MG. The authors measured the volume changes for the transitions among these forms. For U to MG, for example, protein in HCl at pH 2 was loaded into one dilatometer leg, and 0.5*M* KCl which had been brought to pH 2 with HCl was loaded into the other. MG to N involves changes in both pH and salt concentration. Because protein free solvent controls were technically inconvenient for this transition, the triangle of U, MG, and N was expanded to a square whose logic is shown in Figure 3 (modified from Ref. 2). Expansion to a square proved instructive as discussed below.

The four sides of the square constitute a thermodynamic cycle whose steps must add up to zero. Within experimental error, this requirement was met. The results are believed to be the first volumetric cycle demonstrated for protein conformational transitions.

The pH jump experiments (U to N and MG to NCl_X_, where NCl_X_ is N with an unknown number of chloride molecules bound) were accomplished by mixing protein at pH 2 in one leg with a sufficient quantity of MOPS buffer to reach the final pH of 7 in the other leg. After subtracting controls for titration of the MOPS and for protein dilution, the authors computed the volume change for deprotonating the protein itself and transferring its protons to the buffer. This left the volumetric effects of all other processes combined. For U to MG this Δ*V*, +99.6 mL/mol, was attributed primarily to expulsion of water of hydration as the conformation collapsed to the relatively compact MG form. The Δ*V* of N to NCl_X_ of −30.9 mL/mol was surprising. Chloride binding releases electrostricted water molecules, which would lead to a rise in volume. For the volume, change to be negative means that this effect was overwhelmed by another. Small angle x‐ray scattering had shown that salt addition to native cytochrome c reduced the radius of gyration by 1 Å. At pH 7.15 and ionic strength =0.1, the protein, whose pI is 10.0, carries a net charge of +7.1, and its conformation is therefore under a degree of electrostatic stress. The screening brought about by additional KCl would relieve some of this stress, allowing the protein to relax. As the conformations of N and NCl_X_ were not detectably different by CD, the negative volume change was therefore attributed to a reduction in the size and/or number of packing defects. Finally, the number of water molecules expelled from the protein surface in the U to N transition was estimated to have an upper limit of ~600. An interesting approach to estimating the number of hydrating waters has been presented for methane, water itself, and chloride ions^27^ and could be extended to proteins.

Capillary dilatometry usually requires large amounts of protein ranging from a few milligrams to tens of milligrams depending on the protein and the nature of the reaction monitored. Fortunately, in most cases, the protein can be recovered and reused. An alternative technique involves the high precision measurement of solution density with the vibrating tube densimeter.^28^ The cell is a V or U shaped glass tube mounted as a cantilevered beam from its open ends. It is made to vibrate, with the number of vibrations per second depending on the density of the contents. The unit is calibrated with at least two materials of known density, water, and air being the most commonly used. Sample volumes are 1 mL to fill the cell plus several more for inlet tubing and sometimes for rinsing. Extrapolation of protein or nucleic acid concentrations to infinite dilution yields partial specific volumes, from which volume changes are obtained by difference. Among the advantages are smaller volumes than for dilatometry and therefore less material being needed. Densimeters can also be operated as functions of temperature, which is possible but awkward by dilatometry. Accuracy, though, can be a problem. The densities are measured to six or seven significant digits, extrapolated and differences taken, whereas dilatometry gives a direct result. High precision densimeters are also expensive. Nevertheless, both techniques give useful results. The nucleic acid work mentioned above was done using this technique, and it has been used also for proteins.^9^, ^10^

## Conclusions

Volume is an extensive thermodynamic property whose importance in understanding proteins has been emphasized by Kauzmann.^29^ Capillary dilatometry is a way to measure changes in it directly, but technical difficulty has limited its use. The method described here brings it within the capacity of any biochemistry or biophysics laboratory. Insight into the role of hydrating water in reactions involving proteins and nucleic acids thus becomes more readily available.

## Materials and Methods

### *General description of dilatometers and their use*

The dilatometers we use are inverted Y‐tubes as shown in Figure 3. The earliest use of them I could find was by Sreenivasaya and Sreerangachar.^30^ They have come to be called Carlsberg dilatometers because of their extensive use by Linderstrom‐Lang's group at the Carlsberg laboratory (see, e.g., Ref. 31).

One places a carefully measured volume of, for example, a buffered enzyme solution in one leg of the unit and a ligand dissolved in the same buffer in the other leg. An organic solvent not miscible with the aqueous phase is then used to fill the dilatometer, and the capillary is attached. The organic solvent serves as manometric fluid in the capillary. The protein should have been dialyzed against a buffer. It is important that the ligand be made up in buffer from the dialysis so that it will be experience the same solvent conditions as the protein, thereby avoiding spurious volume changes arising from changes due to even slightly different buffer conditions. When working with proteins, in addition, dilution controls are necessary. The volume change of protein dilution against plain buffer and the change of diluting ligand against buffer must both be measured and subtracted from the main experiment. If pH changes are involved, controls lacking protein are necessary as discussed above in the analysis of the refolding of ribonuclease and the MG transitions of cytochrome c. We usually measure the controls at the same time as the main experiment or immediately afterward.

The original units had ground glass joints between the capillary and the dilatometer body. Because of the small diameter of the capillary even small leaks at the joint would compromise the measurement. To avoid leaks each capillary's male joint had to be hand lapped to the dilatometer's female joint with slurries made of successively finer grades of carborundum and finally with jeweler's rouge. Once lapped, capillaries and dilatometer bodies were not interchangeable, and there was always a risk of breakage on opening the unit after an experiment.

The joints had to be greased. The grease at the tip of the joint had to be insoluble in organic solvents, while its top had to have a water insoluble grease, as the dilatometer is thermostated in a water bath. The two greases would meet in the center of the joint when the unit was assembled. The greasing, hand lapping, and other difficulties in handling reduced the usefulness of the technique. Although Katz glued Teflon sleeves to the male joint, thereby eliminating the need for grease, the joints had still to be hand lapped, and the Teflon became scratched easily.^32^ Described below, therefore, is a novel joint which requires no grease, is easy to handle, and allows capillaries and dilatometer bodies to be interchangeable.

#### *The present dilatometers and their use*

**Figure 4:**
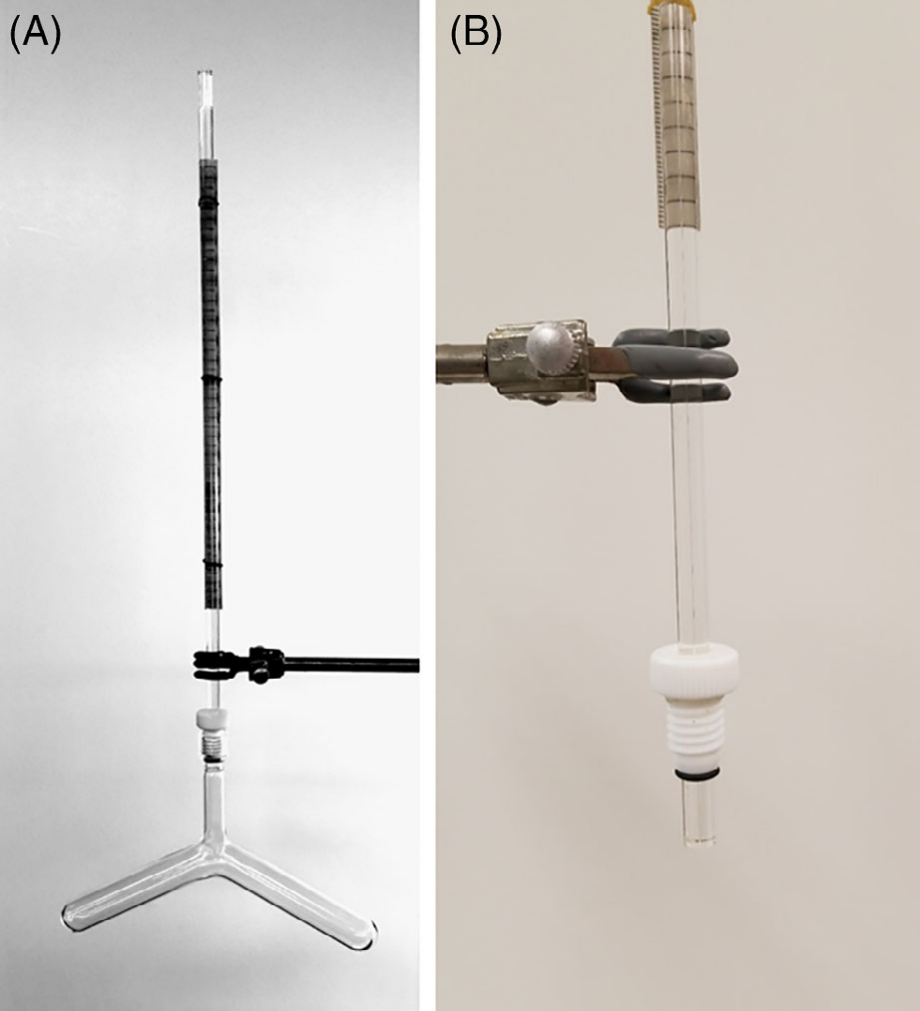
A Carlsberg dilatometer with interchangeable screw‐in capillary. (a) An assembled dilatometer. The unit shown holds 14.7 mL in each leg. (b) A capillary with screw‐in Teflon plug and rubber O‐ring to make the seal. The ruler for measuring the meniscus height is shown in both (a) and (b) (see text).

Dilatometer necks are purchased from Ace Glass, Vineland, NJ. They have screw threads at the top and an indentation just below the threads. A glass blower attaches test tubes of whatever size one wishes to the neck. We have units whose nominal capacities are 5, 15 [Fig. 4(a)], and 33 mL per leg. A solid Teflon (polytetrafluoroethylene) plug whose screw threads match the necks (Ace Glass) is drilled to a diameter slightly smaller than that of the capillary to be used with it. The plug is warmed in hot water to soften it slightly, and the capillary is inserted through it, protruding at the plug bottom by about 1 cm. No grease is necessary. Once the plug has cooled, the capillary is held firmly. A rubber O‐ring is placed over the end of the capillary at the bottom of the plug so that when the joint is mated, the O‐ring forms a seal at the indentation beneath the neck's screw threads (Fig. 4).

Capillaries are purchased from Wilmad Labglass, Vineland, NJ. Our routine size has a bore diameter of 0.008 in. (0.2032 mm) and a nominal calibration of 0.324 μL per centimeter change in meniscus height. We also use capillaries of diameters 0.015 in. (0.3810 mm, 1.140 μL/cm), 0.006 in. (0.1524 mm, 0.182 μL/cm), and 0.004 in. (0.1016 mm, 0.081 μL/cm). Each capillary, however, must be hand calibrated to obtain accurate results. This need only be done once. To calibrate, one fills the working length, typically about 30 cm, with mercury. The capillary and a thermometer calibrated in 0.1°C increments are laid side‐by‐side on the bench. The length of the mercury column is measured, the temperature recorded, and the mercury expelled and weighed. From standard tables of the density of mercury as a function of temperature, the volume is calculated, and from it and the column length, the capillary calibration is obtained. The one shown in Figure 4 and used in the experiment in Figure 1 measures 0.3298 μL/cm change in meniscus height. Once the capillary is calibrated, constancy of the bore is evaluated by moving a 1–2 cm length of mercury to many positions within the capillary and measuring its length. Rejection on these grounds, fortunately, is rare. The manometric fluid, which fills the unit after the aqueous solutions are added, is an organic solvent, originally purified kerosene or toluene. We use high‐pressure liquid chromatography (HPLC) grade heptane, as it is not significantly miscible with the aqueous solutions of interest, and it is pure, relatively inexpensive and relatively nontoxic if handled with care. Organic solvents, however, have coefficients of thermal expansion much greater than that of water. This makes it necessary to immerse the dilatometer body in a precisely controlled water bath whose fluctuations do not exceed one millidegree. Our bath design is described in the Supporting Information.

### *An example experiment*

The following description explains how the dilatometry is done, and it also gives a standard protocol used to teach people the technique. An 8% (w/w) solution of KCl was used. The density of aqueous KCl solutions is tabulated on a w/w basis as functions of concentration and temperature in the ICT.^17^ From those data an 8% (w/w) solution contains 8.4 g/100 mL. This was made up in a 100 mL volumetric flask. Into one leg of the dilatometer, one puts exactly 3 mL of water, while the other leg contains 0.350 mL of the KCl. The unit's legs are then immersed in crushed ice with the neck not in the ice. The HPLC grade heptane to be used is first shaken with water in a separatory funnel to saturate it. When proteins or other biological materials are used, the heptane is shaken with the appropriate aqueous buffer. The unit is filled with the heptane to the point at which it just overflows the neck. After 2–3 min of cooling and topping up with more heptane if necessary, the unit is raised out of the ice, the capillary is firmly and quickly screwed into place, avoiding trapping of air bubbles, and the assembled dilatometer is transferred to the constant temperature bath. It is critical that the bath temperature not vary by more than one millidegree Celsius (Fig. S1)

Most of our experiments are done at 20°C with some at 25°C. Transfer of the dilatometer from the ice to the bath causes the heptane to expand and fill the capillary, overflowing at the top. The overflow is dabbed away gently with a paper towel. Once no further overflow is seen, the unit is left to equilibrate for approximately 30 min. The heptane level at this point will be at the very top of the capillary and must be adjusted to a convenient height for reading. Hold one leg of the unit in the bath for a few seconds. This will warm the unit slightly and will expel some heptane from the top. Remove this gently with a paper towel and let go. The level will drop. Wait another 10–15 min for re‐equilibration before beginning reading.

Expelling heptane requires some experience. If one does not expel enough, the level will be close to the top of the capillary, and should the experiment yield a volume rise, the capillary will overflow. If one expels too much, the level may end up so low that a volume decrease cannot be conveniently read. Even worse, it may plunge all the way down, emptying the capillary and trapping a gas bubble in the dilatometer. The dilatometer must then be removed from the bath, opened, replaced in the ice, and heptane added and the joint re‐mated as described above. With some practice, this can usually be avoided, however. Should the level seem to be falling too quickly, the experiment can usually be saved by grasping a leg of the unit with the hand to warm it. The level will rise. When it gets to the top of the capillary, a Pasteur pipet is used to dispense a drop of heptane onto the top and the hand is removed, allowing the drop to be drawn down into the capillary. More than one drop may be necessary.

### *Reading the meniscus height*

Various ways of measuring the heptane level in the capillary have been used. Kauzmann, for example, used a cathetometer, which I have used on occasion. Normally, however, our lab uses a simple method. An inexpensive clear, transparent ruler with metric gradations along one edge is sliced to remove the other edge, which is in inches. The back of the metric part is spray painted white, and it is attached to the capillary with small rubber bands supplied by a friendly orthodontist (Fig. 4).

In reading the meniscus height through the glass of the capillary, one estimates to 0.1 mm. This takes a little practice, and it is imperative that all readings of a single dilatometer be done by the same person to minimize reading bias. Readings are done typically once a minute and recorded. If two dilatometers are run at the same time, one is read on the minute and the other 30 sec later on the half‐minute. On occasion, we have read a single dilatometer every 15 sec. I have occasionally used four dilatometers at the same time, reading in rotation every 15 sec so that each is read once a minute. One reads until approximately 10 consecutive readings are essentially constant. This establishes the premixing baseline.

### *Mixing*

To mix the two legs, initiating the reaction, one reaches into the tank, grasping the dilatometer by the neck, removing it from its fastener, and rocking to mix the legs. This is done without bringing the unit out of the water bath and with extreme care not to touch the capillary to one's hand or to the edge of the bath, as this may shift it, affecting the later readings. We mix back and forth 10 times, which typically takes about a minute. Readings are resumed until approximately 10 steady data points are obtained.

Protein solutions are often viscous, and the mixing may not yield a uniform postmix solution. To check for this a second mixing is done. If the level returns to the same height as after the first mixing, the experiment is finished. If it does not, further mixing may be needed. With some experiments, several mixings may be necessary before the heptane level stabilizes. In some experiments, mixing is aided by including in the dilatometer a small Teflon covered magnetic stir bar. Rocking the unit back and forth during mixing moves the stir bar from one leg to the other.

Recovery of protein solutions after the experiment is accomplished with a syringe fitted with a piece of tubing long enough to reach the bottom of the dilatometer leg. The protein concentration and, where appropriate, the activity should be remeasured after the experiment. Because the technique is nondestructive, recovered protein can often be reused after reestablishing the initial experimental conditions by dialysis or column methods.

On some occasions, a positive volume change is so large that heptane overflows the top of the capillary, and the experiment is lost. Capillaries with larger bores help in this case. Greater sensitivity, on the other hand, is achieved with narrower bores as described above. The meniscus in bores smaller than 0.004 in. (0.1016 mm), however, is too hard to read and fluctuates too much for reliable results. A specialized capillary whose top was opened out to form a small funnel has been described for use in rare instances when the heptane column breaks due to a large and extremely rapid drop in the meniscus.^1^

### *The constant temperature bath*

The organic solvent used as manometric fluid has a large coefficient of thermal expansion. Because of the narrow bores of the capillaries, even small temperature fluctuations cause the meniscus height to vary too much for reliable reading. Stability of the constant temperature bath is therefore critical, with variations not exceeding one millidegree. The design of our bath is provided in the Supporting Information as an example.

## Conflict of Interest

The author has no competing interests.

## Supporting information


**Appendix S1:** Supplementary Information

**Supplementary Figure S1** Constant temperature bath. (A) The four heating lights are on. The copper tubing through which cooling water circulates is near the bottom of the bath. It is bent so that is sits above the bottom of the bath to improve mixing and thereby constancy of temperature. The two stirring motors drive water down toward the bottom of the tank. (B) The heating lights are off. When the bath is equilibrated the lights are on and off for the same duration. The bath should not be used if persons subject to epileptic seizures are present, as the rhythmic flashing can cause a seizure. Some details of dilatometer attachment are shown, and one of the sockets for a light is visible at the left.
