# Supplementary material for: The measurement of volume change by capillary dilatometry
Source: Protein Sci. 2019 Apr 29;28(6):1135–42. doi: 10.1002/pro.3626 (PMC6511832; doi:10.1002/pro.3626)
Supplement: Supplementary file 1 — Appendix S1: Supplementary Information [file PRO-28-1135-s001.docx]

**Supplementary Material – The Constant Temperature Bath**

Dilatometry requires a high degree of stability in the operating temperature. Heptane, like most hydrocarbon solvents, has a coefficient of thermal expansion much greater than that of water. Because the capillary diameters are small, even small fluctuations in the temperature cause the meniscus height in the capillary to fluctuate excessively. Temperature must therefore be stable to within one millidegree. Described here is the bath we have built to meet this requirement.

Its core is a 75 gallon aquarium. In hindsight it could have been smaller. A capacity of 35-50 gallons would have served, but when we built it, we anticipated using many dilatometers at the same time. The tank is insulated on its back, sides and bottom with 2.5 inches (6.35 cm) of Styrofoam. The front has a one-half inch (1.27 cm) thick sheet of transparent plexiglass set off from the front of the tank by a one inch (2.54 cm) rim of Styrofoam. The Styrofoam front rim is attached to the sides by glue reinforced with wooden dowel rods which are glued in place. The use of heat conducting metal is thereby avoided.

Temperature control involves continuous cooling with intermittent heating. The cooling is supplied through the copper tubing, shown in Figure S1A, whose diameter is 0.5 inch (1.27 cm). The tubing is bent so that it is set off from the tank bottom to aid in thorough mixing of the water. The mixing is provided by two electric stirrers. When the tank temperature is set to 20 ⁰C, our most common operating temperature, the water circulating through the tubing is set to 19 ⁰C. It is supplied by a Haake Model A81 unit which is not seen in the figure.

Heating is supplied by four 150 watt incandescent light bulbs which are mounted in waterproof sockets attached to a plywood board that covers the rear third of the tank surface. The board is reinforced across its entire length with an aluminum angle bracket. Temperature control is by a Tronac PTC-41 controller. The thermal sensor is suspended in the tank from one of the dilatometer attachment devices. Light bulbs are used because they have practically no thermal lag, becoming hot and cool within a few seconds of being turned on or off. They are on in Figure S1A and off in S1B.

One of the two motor driven stirring units is partly visible in Figure S1B. It is black. It is imperative that vibrations be minimized. To this end, the propeller shafts have been carefully straightened in a machine shop. In addition, the motors are not attached to the tank table; they are suspended independently, in our case on the wall behind the tank.

Dilatometers are held in place by high technology attachment devices screwed to a one inch (2.54 cm) thick plexiglass sheet that runs across the middle third of the tank surface. Like the mounting board for the stirrers, it is reinforced across its length by an aluminum angle bracket. Dilatometers are held by their capillaries so the entire dilatometer body and a centimeters or two of the capillary are under the water.

From the time the system is turned on it typically takes 30-45 minutes to equilibrate at 20 ⁰C. The time can be shortened by adding ice or hot water as needed. Once stabilized, the meniscus level is quite sensitive to small temperature fluctuations, as mentioned above. Simply placing the hand in the tank near the dilatometer will add enough heat to cause the meniscus to rise.

Supplementary Figure S1: Figure Legend, Supplementary Material

Constant temperature bath. (A) The four heating lights are on. The copper tubing through which cooling water circulates is near the bottom of the bath. It is bent so that is sits above the bottom of the bath to improve mixing and thereby constancy of temperature. The two stirring motors drive water down toward the bottom of the tank. (B) The heating lights are off. When the bath is equilibrated the lights are on and off for the same duration. The bath should not be used if persons subject to epileptic seizures are present, as the rhythmic flashing can cause a seizure. Some details of dilatometer attachment are shown, and one of the sockets for a light is visible at the left.
