# Supplementary figures and images for: The measurement of volume change by capillary dilatometry
Source: Protein Sci. 2019 Apr 29;28(6):1135–42. doi: 10.1002/pro.3626 (PMC6511832; doi:10.1002/pro.3626)

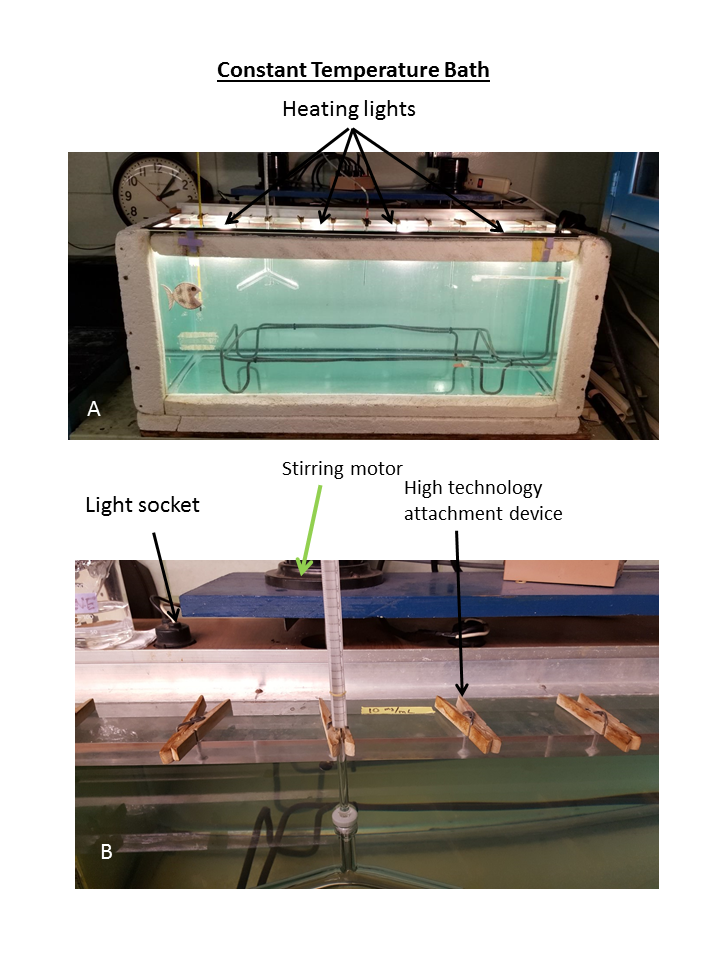

Supplement: Supplementary file 2 — Supplementary Figure S1 Constant temperature bath. (A) The four heating lights are on. The copper tubing through which cooling water circulates is near the bottom of the bath. It is bent so that is sits above the bottom of the bath to improve mixing and thereby constancy of temperature. The two stirring motors drive water down toward the bottom of the tank. (B) The heating lights are off. When the bath is equilibrated the lights are on and off for the same duration. The bath should not be used if persons subject to epileptic seizures are present, as the rhythmic flashing can cause a seizure. Some details of dilatometer attachment are shown, and one of the sockets for a light is visible at the left. [file PRO-28-1135-s002.tif]
